# Supplementary material for: Firing feature-driven neural circuits with scalable memristive neurons for robotic obstacle avoidance
Source: Nat Commun. 2024 May 21;15:4318. doi: 10.1038/s41467-024-48399-7 (PMC11109161; doi:10.1038/s41467-024-48399-7)
Supplement: Supplementary file 1 — Supplementary Information [file 41467_2024_48399_MOESM1_ESM.pdf]

Supplementary Materials for

Firing Features-Driven Neural Circuits with Scalable Memristive Neurons for Robotic Obstacle Avoidance

Yang et al.

The file includes:

Supplementary Information 1. Construction of the device model with switching voltage stochasticity and the H-H circuit model with the probabilistic transition.

Supplementary Figure 1. Structural diagram and SEM characterization of the device.

Supplementary Figure 2. Schematic of the H-H neuron generating a single peak in spiking.

Supplementary Figure 3. Schematic of the H-H neuron generating double peaks in bursting.

Supplementary Figure 4. Response of the H-H neuron circuit receiving negative inputs.

Supplementary Figure 5. The 23 biological neuron firing behaviors demonstrated in the H-H neuron based on the NbO_2 devices.

Supplementary Figure 6. The firing behaviors of the H-H neuron circuit output adjusted through increasing the input voltage under fixed circuit parameters in the simulation.

Supplementary Figure 7 The firing feature transition of the neuron circuit output is reproducible and reversible.

Supplementary Figure 8. Schematic of the device threshold voltage measurement using an oscillating circuit.

Supplementary Figure 9. Normal Q-Q plot of threshold distribution statistics for NbO_2 devices used in H-H neuron circuits.

Supplementary Figure 10 The effect of high and low resistance randomness of the devices on the circuit output.

Supplementary Figure 11. Responses of the H-H neuron circuit to the input with 500 μs pulse width, 0.3-1.5 V amplitude input with a step rate of 0.1 V.

Supplementary Figure 12. The curves of the outputs' interspike interval as a function of the input.

Supplementary Figure 13. Schematic illustration of JISI plot.

Supplementary Figure 14. Schematic of selective communication scheme in biological neurons.

Supplementary Figure 15. The selective communication output of the neural circuits under different input voltage.

Supplementary Figure 16. The device model with the randomness of the V_{TH} and V_{Hold}.

Supplementary Figure 17. The selective communication output of the neural circuits under different input voltage.

Supplementary Figure 18. Flow chart of robot obstacle avoidance behavior control.

Supplementary Figure 19. A rendering of the robot's obstacle avoidance.

Supplementary Figure 20. Simulated latency scaling of the memristive H-H neurons.

Supplementary Figure 21. Simulated energy scaling of the memristive H-H neurons.

Supplementary Figure 22. Comparison of the scalability of the memristor-based on-chip LIF / H-H neuron circuits with CMOS technologies.

Supplementary Figure 23. The SCNC was constructed via a printed circuit board (PCB) and a breadboard.
Supplementary Table 1. Comparison with literature reported artificial neurons based on memristors.
Supplementary Table 2. Circuit parameters used for achieving 23 firing behaviors of the H-H neuron.
Supplementary Table 3. Circuit parameters used for experiment or simulation in the neural circuits.
Supplementary Table 4. Comparison with literature reported artificial neurons based on CMOS.
Supplementary Table 5. Parameters used for SPICE model.

Supplementary Information 1. | Construction of the device model with switching voltage stochasticity and the H-H circuit model with the probabilistic transition.

The device model with switching voltage stochasticity was constructed based on an empirical model that matches the measured data well (fig. S15(A)). The empirical model is described by a piecewise function:

$$R_{High}=a\cdot e^{bv}+c\cdot e^{dv} \tag{1}$$

$$R_{Low}=f\cdot e^{gv}+h\cdot e^{iv} \tag{2}$$

where R_{High} and R_{Low} are the HRS and LRS of the device, respectively. V is the input voltage and a,b,c,d,f,g,h and i are fitting parameters. The initial state of the device follows Eq.1 and is in HRS. Once the voltage on the device exceeds the V_{TH}, the device switches to the LRS and follows Eq.2 then. When the voltage on the device is less than V_{Hold}, the device switches back to the HRS from LRS, and again follows Eq.1. In order to further construct the device model with threshold randomness, V_{TH} and V_{Hold} values of the empirical model are randomly taken within a range that conforms to a Gaussian distribution, whose mean and variance value are based on experimental data, rather than a constant value, as shown in Figure S16(b).

The simulated H-H neuron circuit consists of two NbO_2 devices, two resistors, two capacitors and two voltage sources. The behavior of the whole H-H neuron circuit can be described by four coupled first-order ordinary differential equations, which are derived by using Kirchhoff's voltage law (KVL) and Kirchhoff's current law (KCL). The equations are rewritten as:

$$\frac{dV_{Na}}{dt}=\frac{1}{C_1}\cdot\left(\frac{V_{in}-V_{Na}}{R_1}-i_{Na}-\frac{V_{Na}-V_K}{R_2}\right) \tag{3}$$

$$\frac{dV_K}{dt}=\frac{1}{C_2}\cdot\left(\frac{V_{Na}-V_k}{R_2}+i_K\right) \tag{4}$$

$$i_{Na}=\frac{V_{Na}-E_1}{R_{TS1}} \tag{5}$$

$$i_K=\frac{E_2-V_K}{R_{TS2}} \tag{6}$$

where C_1, C_2, R_1, R_2, E_1 and E_2 are the capacitors, resistors and voltage sources in the H-H neuron circuit, respectively. V_{Na} is the voltage at the node between R_1 and R_2, which is the voltage at the top electrode of TS_1. V_K is the voltage at the bottom electrode of TS_2, which is also the output of the whole H-H neuron circuit. Introducing the device model with threshold randomness into the H-H neuron circuit model, we can obtain the output with the probabilistic transition between bursting and spiking modes, which is consistent with the experiment data.

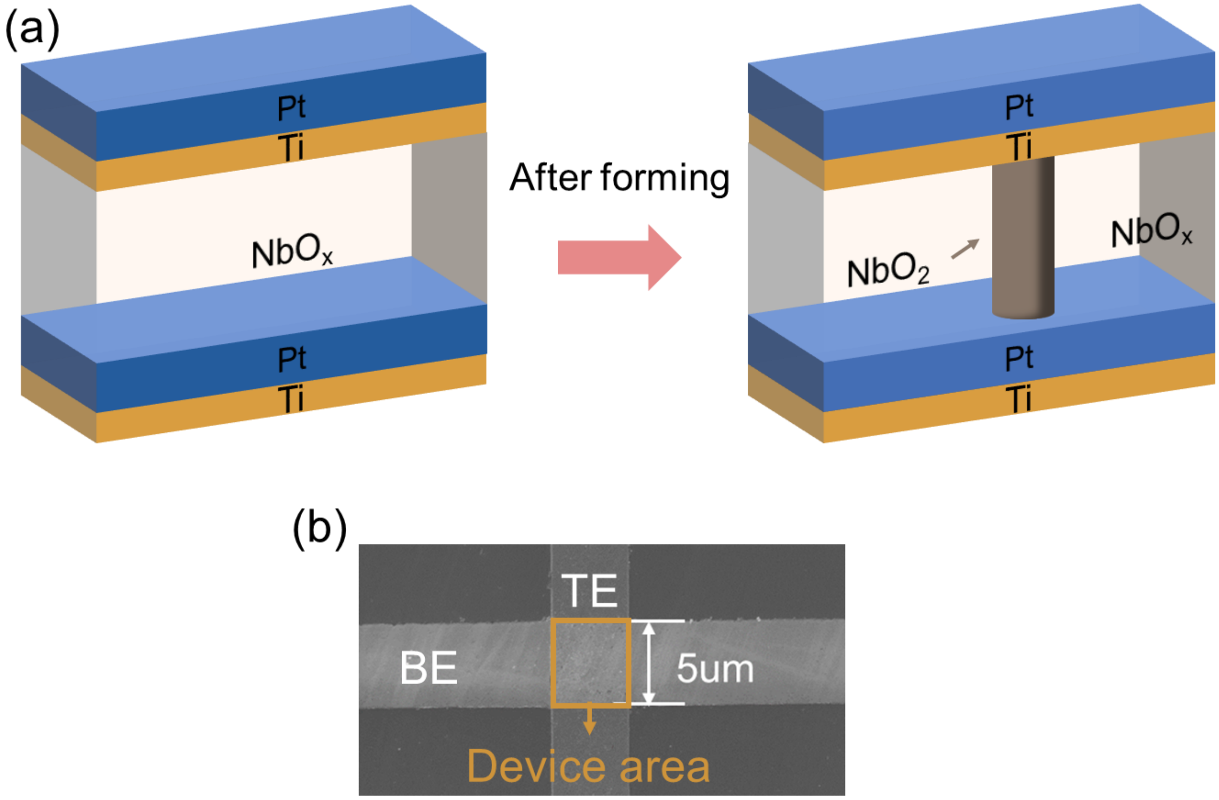

Supplementary Figure 1. Structural diagram and SEM characterization of the device. a) Schematic diagram of NbO_2 device structure. The used device for building neuronal circuits is with a Ti/Pt-NbO_2-Ti/Pt sandwich structure. The device requires an electroforming process to form a conductive NbO_2 filament, after which the device features typical volatile threshold switching behavior. b) The scanning electron microscope of the NbO_2 device. The NbO_2 device has an area of 5 μm*5 μm.

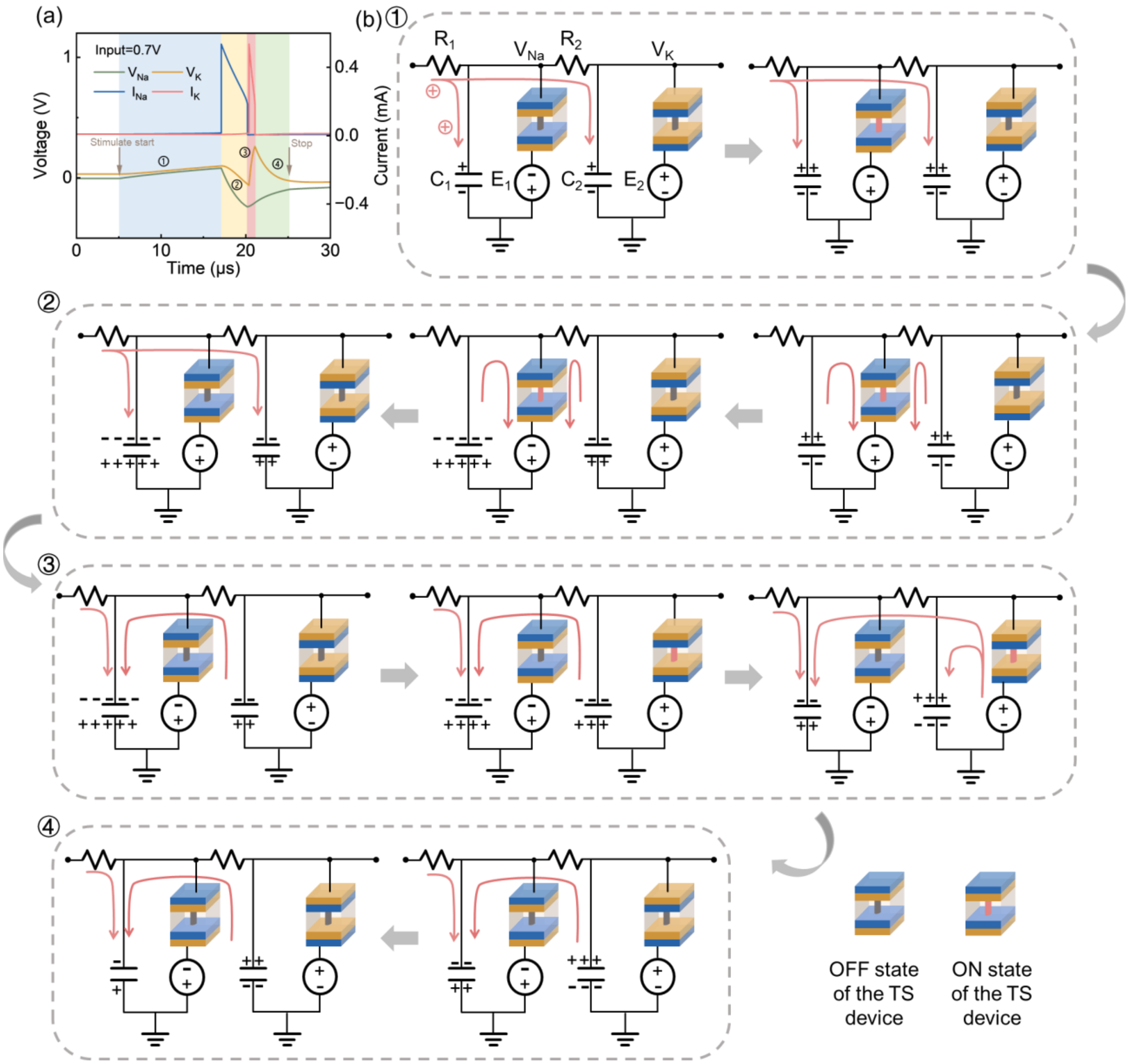

Supplementary Figure 2. Schematic of the H-H neuron generating a single peak in spiking. a) The complete process of the circuit to generate a single peak output is divided into four stages. When there is no input to the neuronal circuit, the two TS devices are both in the HRS, which means Na^+ and K^+ channels are closed, corresponding to the resting state of the biological neuron. b) ① When the input is applied to the circuit, the two capacitors start to integrate, resulting in gradually increasing V_{Na} and V_K. TS_1 switches on when the voltage reaches the V_{TH} of TS_1, manifested by a sudden increase of the current flowing through the TS_1. ② The resistance of TS_1 drops dramatically, and the voltage on C_1 is pulled down towards the negative constant voltage source. Then, C_1 discharges through TS_1 by the negative source, and the V_{Na} decreases, inducing the gradual decrease of the voltage on TS_1, and eventually, the TS_1 turns off when the

voltage on it is below V_{Hold}. ③ C_1 discharges through R_2, the V_K coupled to the V_{Na} gradually decreases, resulting in a gradual increase of the voltage on TS_2 until, and TS_2 switches on when V_{TH} is reached, the current through TS_2 increases rapidly. Once the TS_2 turns on, C_2 is pulled up towards the positive constant voltage source through TS_2, causing V_{Na} and V_K gradually increase. ④ As V_K increases, the voltage on TS_2 decreases and finally turns off. V_{Na} and V_K gradually return to the resting state and prepare for the next firing. So far, both TS devices complete an on-off behavior and the neuron circuit fires a spiking action potential. In this case, the TS_2's switching on is behind the TS_1's switching off.

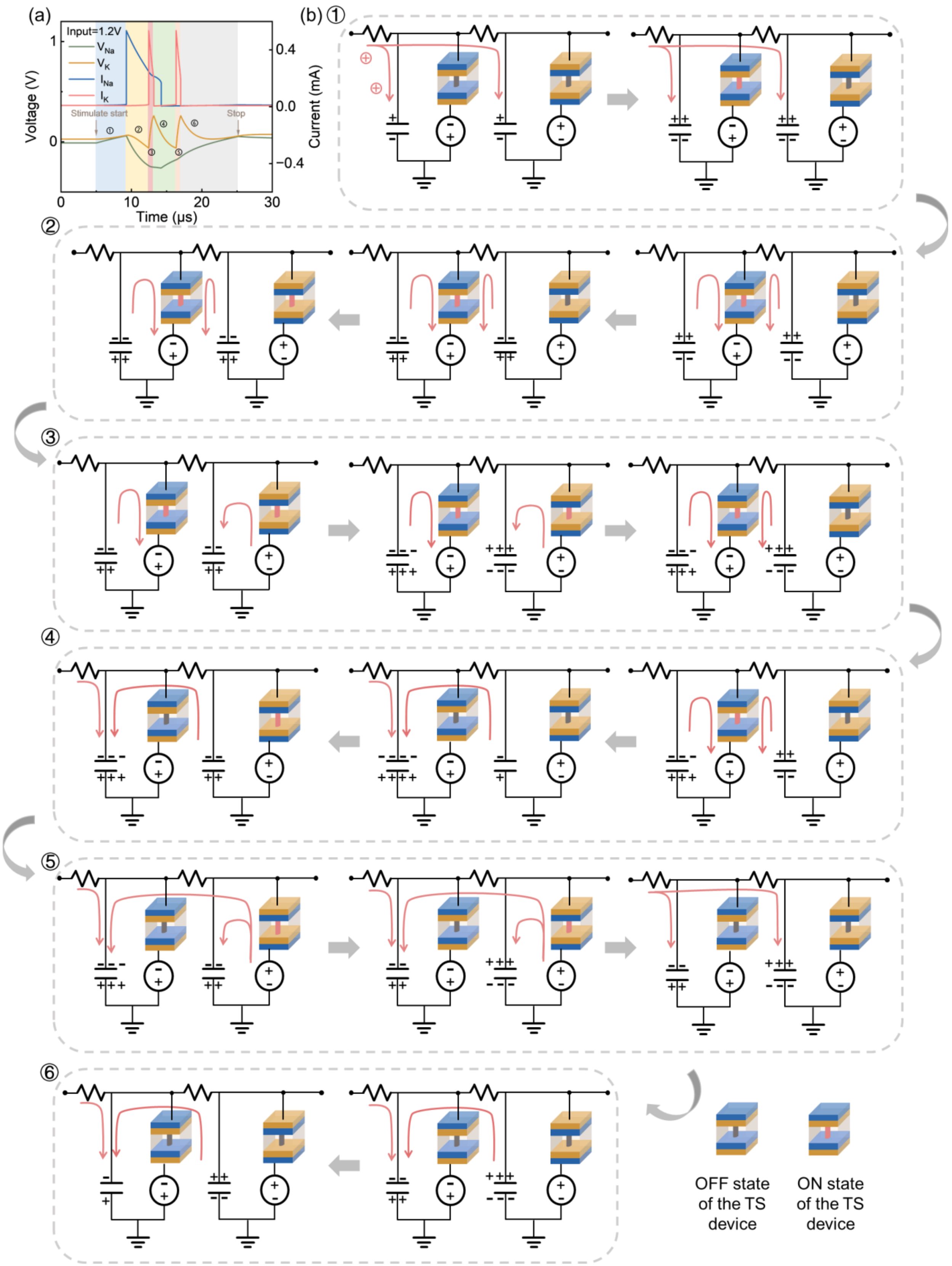

Supplementary Figure 3. Schematic of the H-H neuron generating double peaks in bursting. a) The complete process of the circuit to generate double peaks output can be divided into six stages. When there is no input to the neuronal circuit, the two TS devices are both in the HRS, which means Na^+ and K^+

channels are closed, corresponding to the resting state of the biological neuron. b) ① Same as ① in Supplementary Figure 2. ② The resistance of TS_1 drops dramatically, and the voltage on C_1 is pulled down towards the negative constant voltage source. Then, C_1 charges through TS_1 by the negative source, and the V_{Na} decreases. The V_K coupled to the V_{Na} through R_2 also gradually decreases, which results in an increasing voltage on the TS_2 and then the switching on of the TS_2. ③ Once the TS_2 turns on, C_2 is pulled up towards the positive constant voltage source through TS_2, which leads to an increase in V_K and slows down the rate at which the negative source charges C1, thus slowing down the rate at which V_{Na} decreases and prolongs the on time of TS_1. As V_K increases, the voltage on TS_2 decreases and finally turns off. ④ After TS_2 switches off, C_1 and C_2 continue to be charged by the negative source through the on-state TS_1, and V_{Na} and V_K continue to decrease until the voltage on TS_1 is small enough to V_{Hold} and TS_1 switches off. After TS_1 switches off, C_1 discharges through R_2, V_{Na} increases and V_K decreases. The voltage drop across TS_2 increases to the V_{TH} of the TS_2, leading to the second switching on of the TS_2. ⑤ Same as ③ in Supplementary Figure 2. ⑥ Same as ④ in Supplementary Figure 2. So far, both TS devices complete an on-off behavior and the neuron circuit fires two action potentials in bursting. In this case, the TS_2's switching on is before the TS_1's switching off.

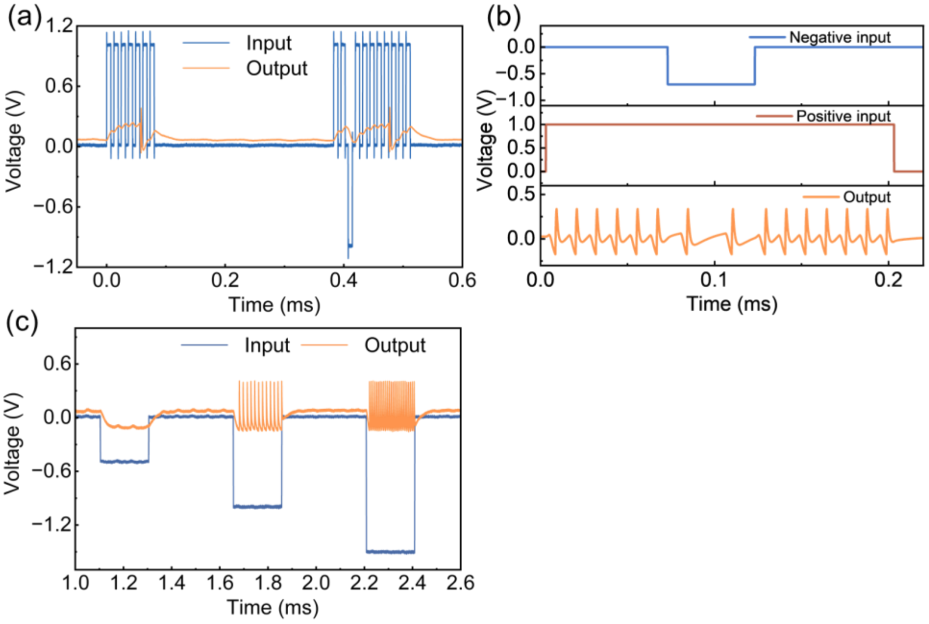

Supplementary Figure 4. Response of the H-H neuron circuit receiving negative inputs. (a) For pulse input with short pulse width and a small negative amplitude, it slows down the process of membrane potential integration and hinders the firing of spikes. (b) For persistent input, inputs with values smaller than the negative threshold do not cause spiking firing, while values larger than the threshold do. The output frequency still increases with the absolute value of the input amplitude. (c) For integration of positive and negative inputs, when the H-H neuron receives a 1.0 V positive input, it fires at a higher frequency. While when the H-H neuron receives a - 0.7 V voltage at the same time, the firing frequency will be significantly reduced.

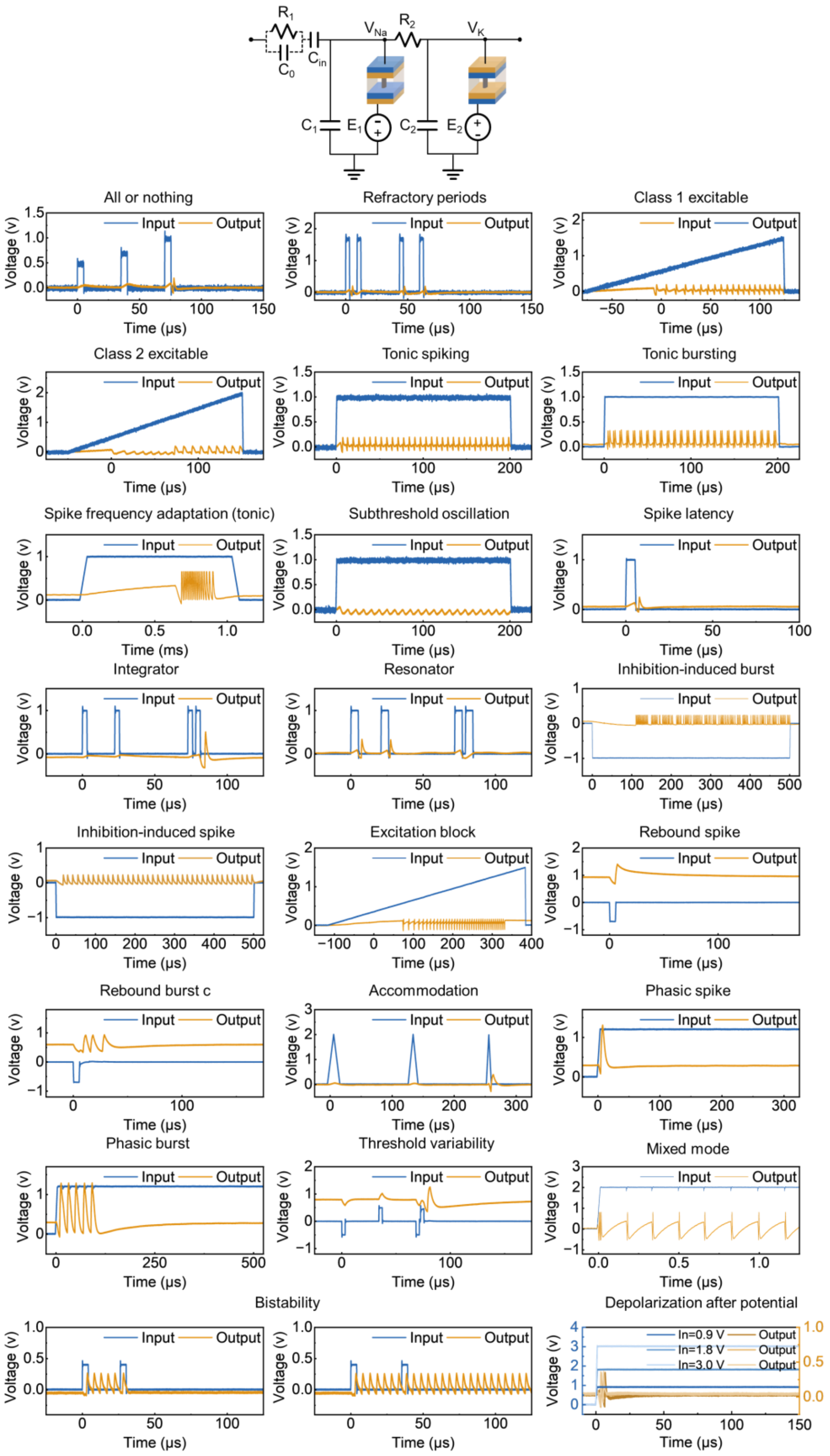

Supplementary Figure 5. The 23 biological neuron firing behaviors demonstrated in the H-H neuron based on the NbO_2 devices. The circuit parameters for different firing behavior are shown in Table S4.

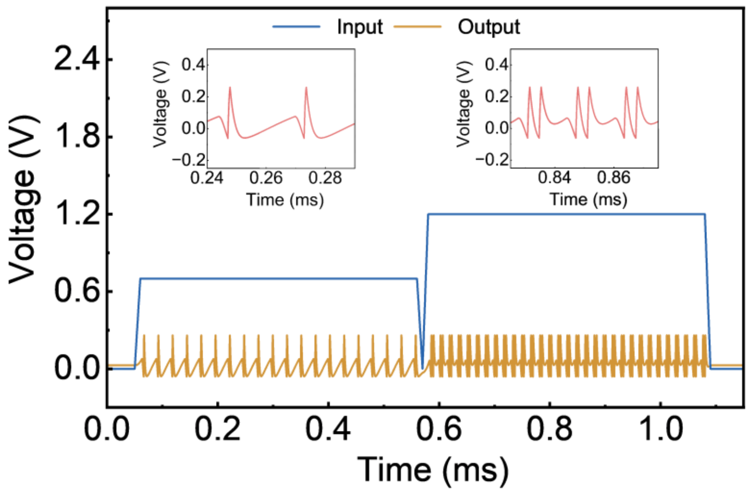

Supplementary Figure 6. The firing behaviors of the H-H neuron circuit output can be adjusted through increasing the input voltage under fixed circuit parameters in the simulation. The firing behavior is in spiking when the input is 0.7 V, while is in bursting when the input voltage is 1.2 V.

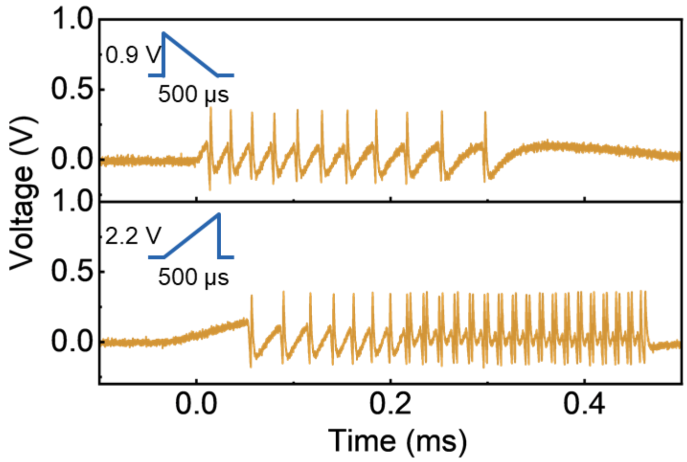

Supplementary Figure 7. The firing feature transition of the neuron circuit output is reproducible and reversible. When the input is gradually decreased from 0.9 V to 0, the output is in spiking all the time but the instantaneous frequency is gradually decreased. When the input gradually increases from 0 V to 2.2 V, the output is initially in the spiking feature, and changes to the bursting feature when the input is larger than 1.0 V, which is consistent with the law of Fig.2e.

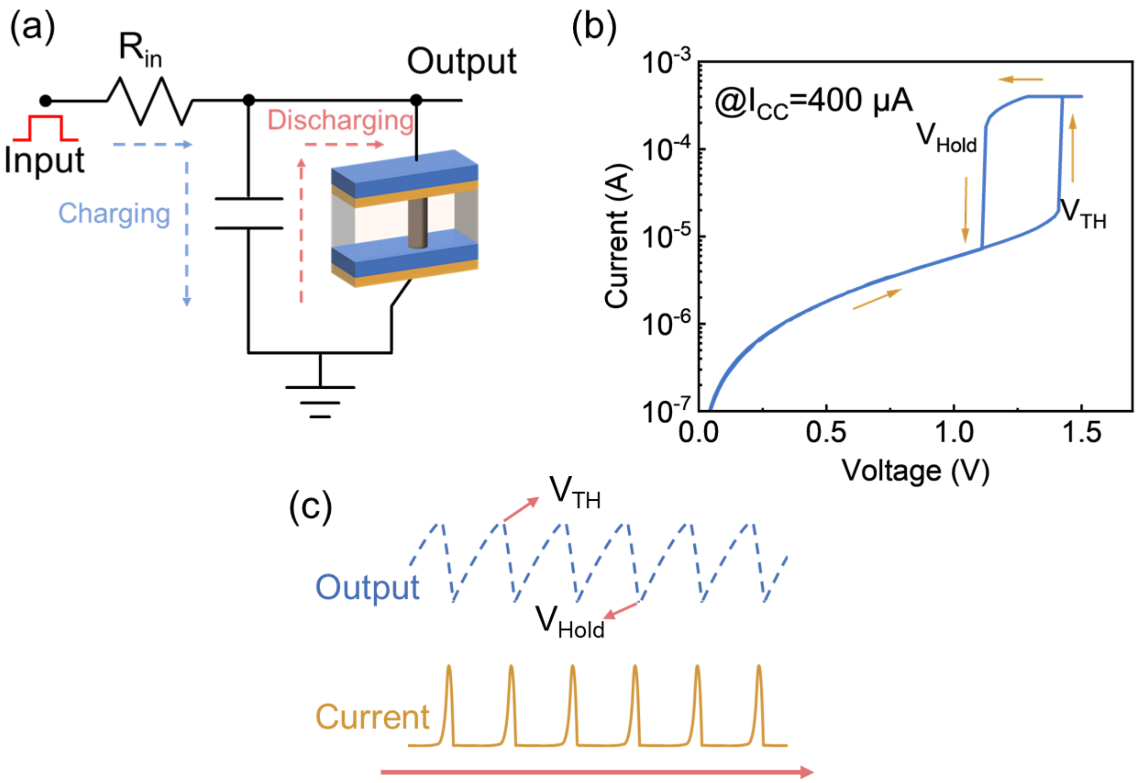

Supplementary Figure 8. Schematic of the device threshold voltage measurement using an oscillating circuit. a) Schematic diagram of the oscillation circuit used to measure the threshold voltage of the NbO_2 memristor devices. The circuit consists of a resistor and a NbO_2 device with the parasitic capacitor. b) Threshold switching characteristics of devices. In DC scanning, when the voltage applied to the device exceeds the threshold voltage (V_{TH}), the device will switch from the high resistance state (HRS) to the low resistance state (LRS) at 400 μA compliance current and back to the HRS when the voltage applied to the device is less than the hold voltage (V_{Hold}). c) Schematic of voltage output in (a) under a fixed voltage input. When the input voltage is applied, the capacitor charges, and the voltage on the NbO_2 device gradually increases until reaches the V_{TH}, which makes the device switch to LRS. As the device switches on, the capacitor discharges, and the voltage on the device decreases until the voltage on the device is less than V_{Hold}, the device switches off and the capacitor starts charging again. So far, an oscillation is completed. Therefore, the maximum and minimum values of the circuit output are the V_{TH} and V_{Hold} values of the device.

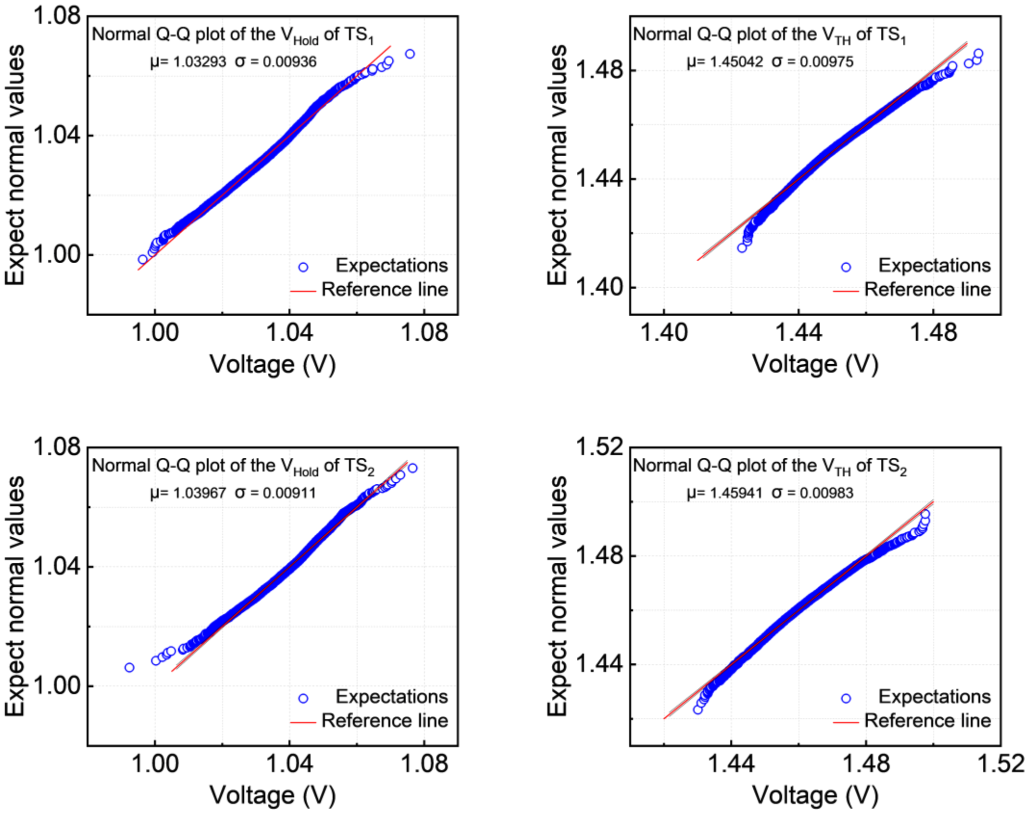

Supplementary Figure 9. Normal Q-Q plot of threshold distribution statistics for NbO_2 devices used in H-H neuron circuits. The V_{TH} and V_{Hold} of the devices satisfy the Gaussian distribution.

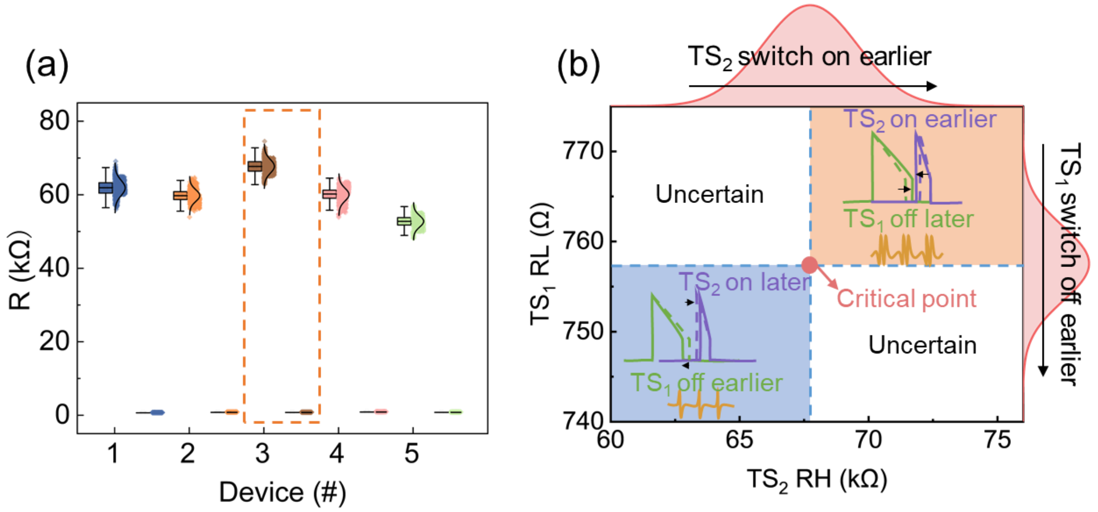

Supplementary Figure 10. The effect of high and low resistance randomness of the devices on the circuit output. (a) The distribution of the high and low resistance values of the five devices under 1000 cycles. When the voltage on the device approaches V_{TH}, the high resistance exhibits fluctuations ranging from 10 K to 20 K, while the low resistance fluctuates within a range of 20 Ω to 50 Ω across different cycles. (b) The influence of TS1 low resistance and TS_2 high resistance on the output. when the low resistance of TS1 is larger, the on time of TS_1 lasts longer, which is caused by the slower discharge in C_1. As mentioned in the main text, when the switching-off time of TS_1 is delayed, the circuit will tend to produce bursting firing behavior. However, when the high resistance of TS_2 becomes larger, it will lead to a smaller voltage reduction at V_K in the initial state of the circuit, which means the voltage on TS_2 is closer to V_{TH} of TS_2.Hence, though the lager high resistance will cause the C_2 to charge slower, the lower initial voltage will still cause the device TS_2 to turn on earlier. Therefore, when the low resistance of TS_1 is larger and the high resistance of TS_2 is larger, the device is more likely to produce bursting behavior under the same input, otherwise it will produce tonic spiking behavior.

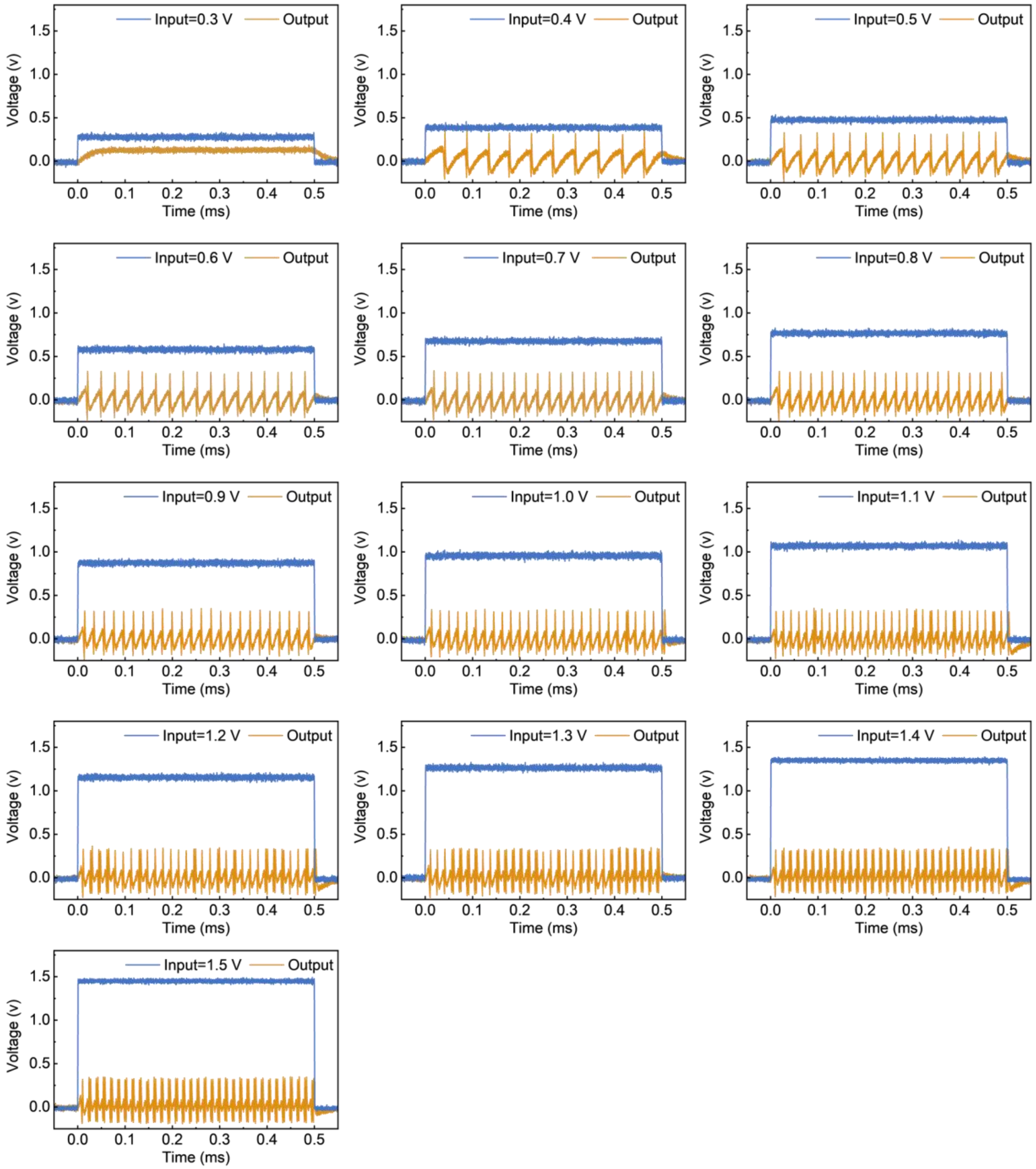

Supplementary Figure 11. Responses of the H-H neuron circuit to the input with 500 µs pulse width, 0.3-1.5 V amplitude input with a step rate of 0.1 V. The circuit shows no spike under the input of 0.3 V, for the input of 0.3 V is not enough to make the voltage on TS_1 reach the V_{TH}. When the input is 0.4-0.9 V, the neuron fires in the spiking feature. When the input increases to 1.0 V, bursts begin to appear in the output of the circuit, showing a mixed firing behavior. With the increase of the input, the probability of bursting gradually increases, until the input reaches 1.5 V, and the spikes in output are all in the bursting

feature. It is worth noting that the first spike of the output with a 1.5 V pulse is in the spiking feature due to the rising edge of the pulse, which will not exist for continuous input.

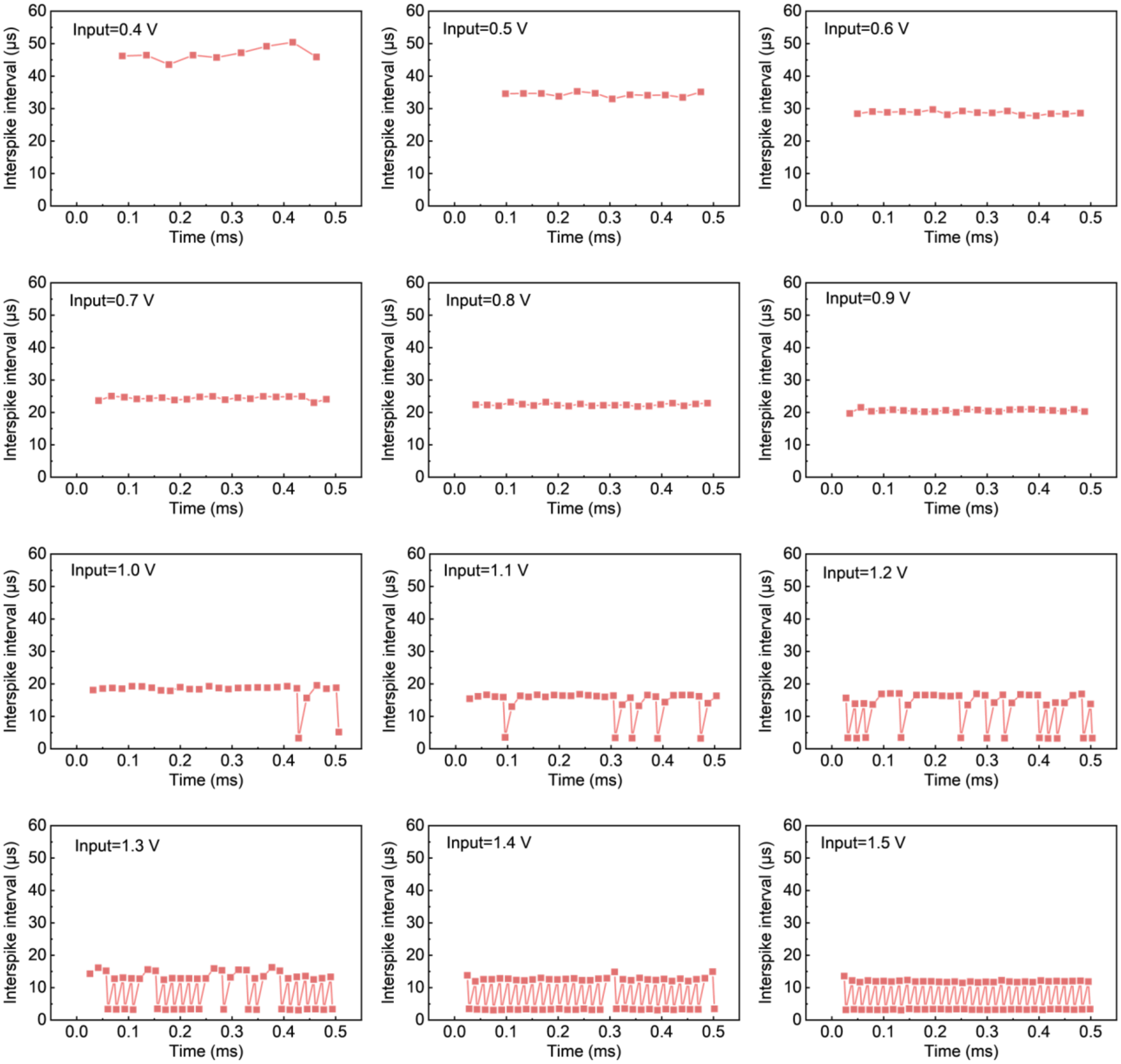

Supplementary Figure 12. The curves of the outputs' interspike interval as a function of the input. For the spiking feature, the circuit fires spikes at almost constant firing rates, so the interspike interval curves are close to straight lines. For the mixed firing features, the interspike interval curves exhibit irregular oscillations, where the larger value represents the spikes in the spiking features in the mixed pattern or the first peaks in bursts, and the smaller value represents the second peaks in bursts. When the output is fully in bursting, the interspike interval curve appears as a regular oscillation.

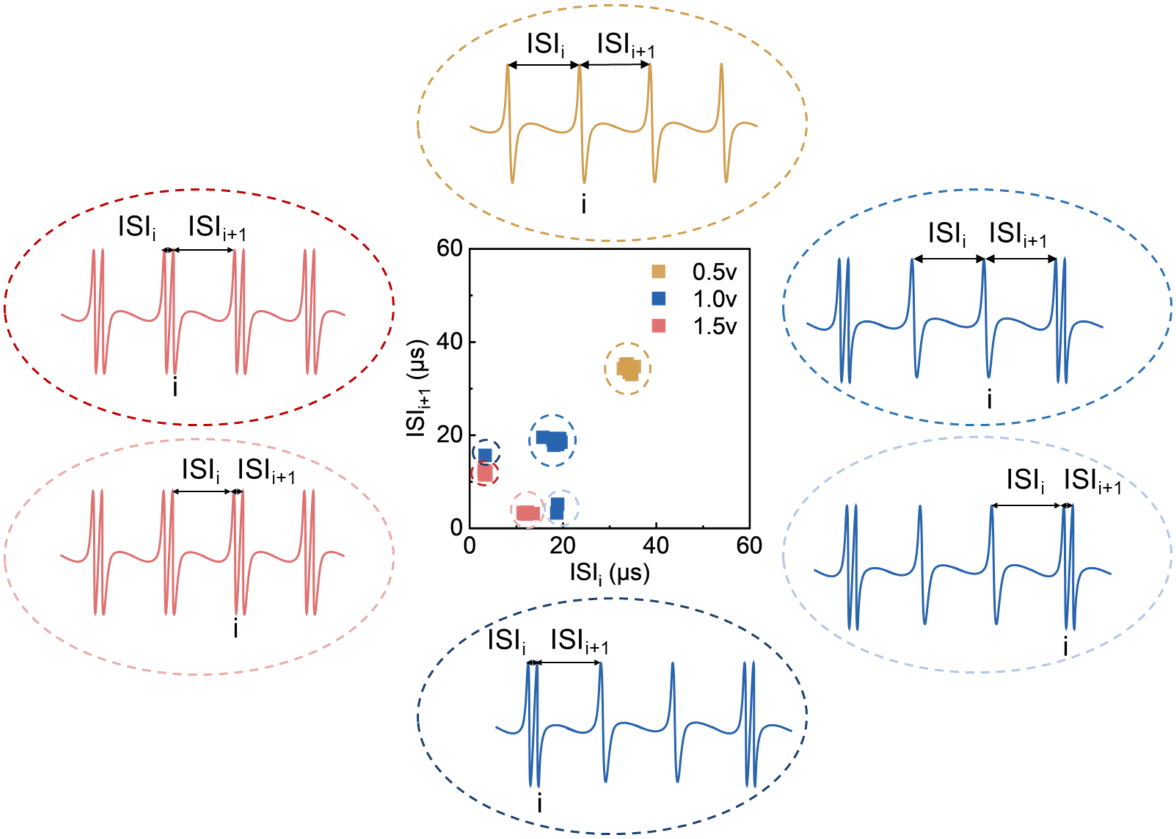

Supplementary Figure 13. Schematic illustration of JISI plot. For the spiking feature (yellow points), the interval between spikes is basically unchanged, so that ISIs points are gathered in the diagonal position. For the bursting feature (pink points), the ISIs points are distributed along the coordinate axis. ISIs of the first spikes in the bursts are closer to the X-axis, while the second spike is closer to the Y-axis. For mixed firing features (blue points), ISIs are located partly on the diagonal (spikes in spiking) and partly in the region close to the coordinate axis (spikes in bursting). Therefore, we can directly obtain the characteristics of the neuron output for different inputs from a JISI plot.

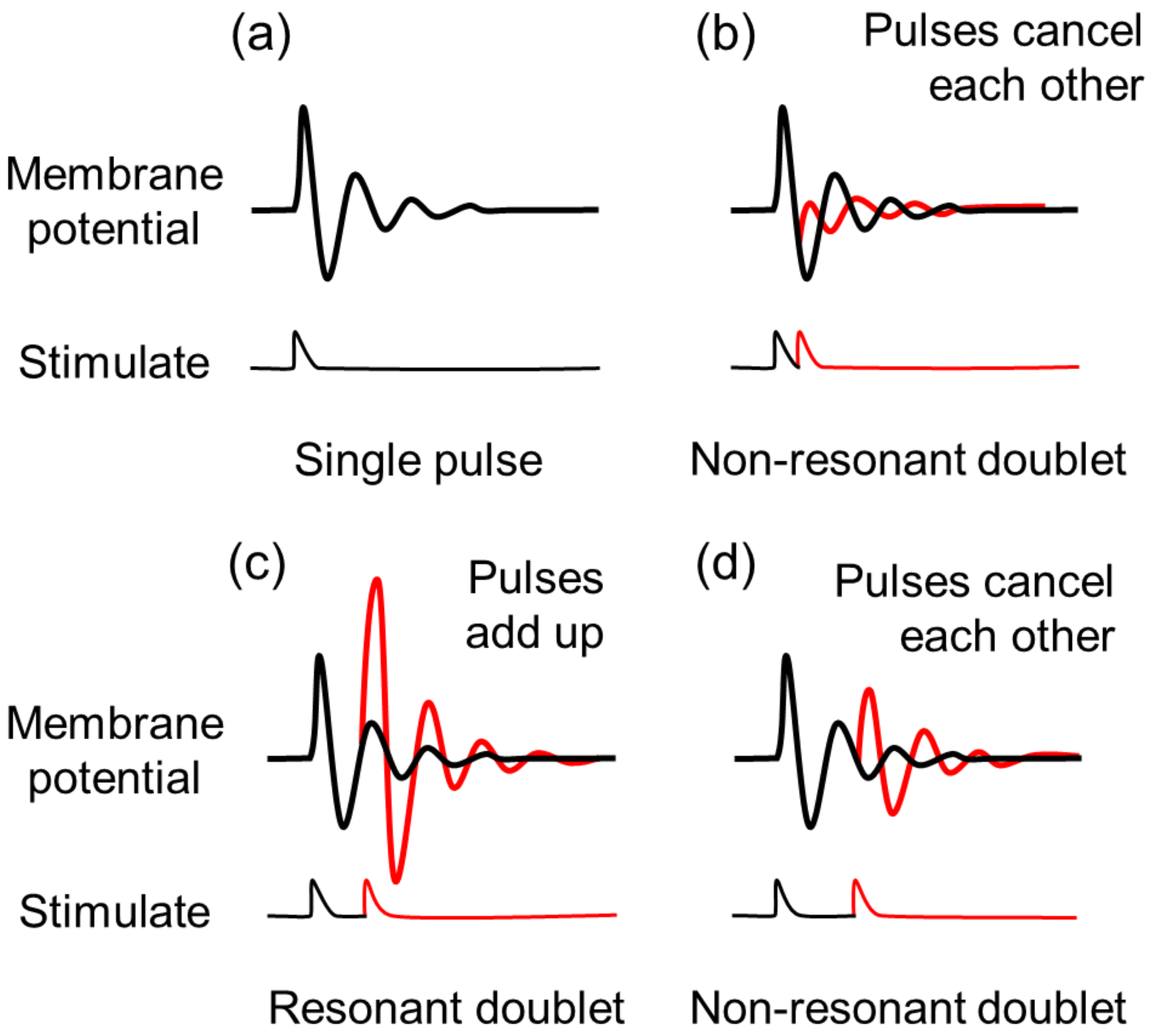

Supplementary Figure 14. Schematic of selective communication scheme in biological neurons. a) Some neurons exhibit subthreshold membrane potential oscillations when stimulated by brief synaptic input or an injected pulse of current. The effect of the second spike depends on its timing relative to the first spike. b) If the interval between pulses is near half the natural period, the second pulse arrives during the falling phase of the oscillation, and it leads to a decrease in oscillation amplitude. c) If the interval between the spikes is near the natural period, the second spike arrives during the rising phase of the oscillation, and it increases the amplitude of the oscillation even further. d) Similarly, the spikes cancel each other out when the inter-pulse period is 60% greater than the natural period[1].

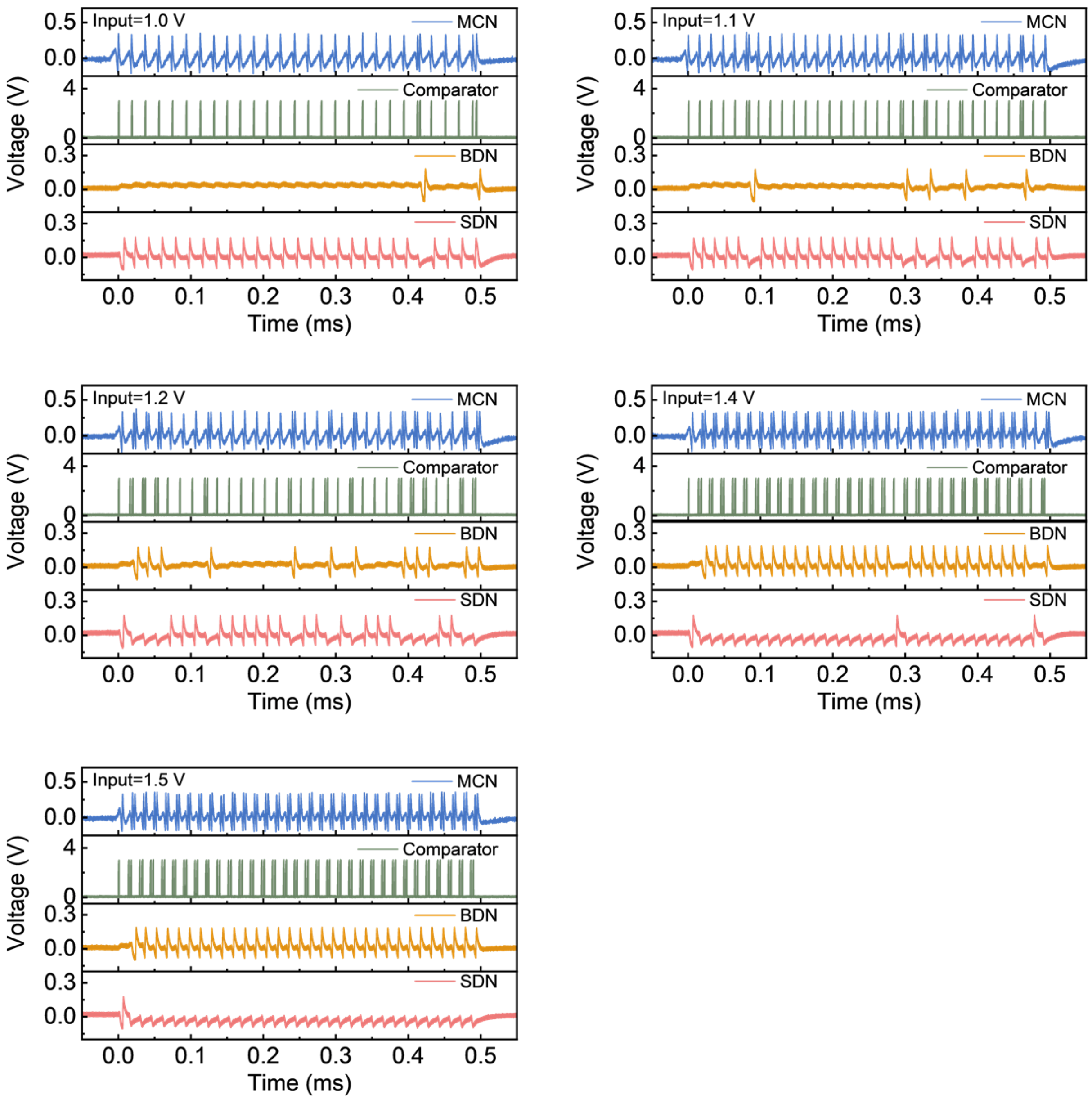

Supplementary Figure 15. The selective communication output of the neural circuits under different input voltage. When the stimuli input is applied, the mixed-coded neuron (MCN) fires with a mixed pattern due to the probabilistic feature transition (blue panel). Then such a mixed spike train is transmitted to both the bursting-detection neuron and spiking-detection neuron through the comparator (green panel). The bursting detection neuron (BDN) fires only when a bursting event happens (yellow panel), while the spiking detection neuron (SDN) only responds to the spiking event (pink panel). As the input increases, the bursting probability in MCN gradually increases, so the output frequency of the bursting-detection neuron gradually increases and the output frequency of the spiking-detection neuron gradually decreases.

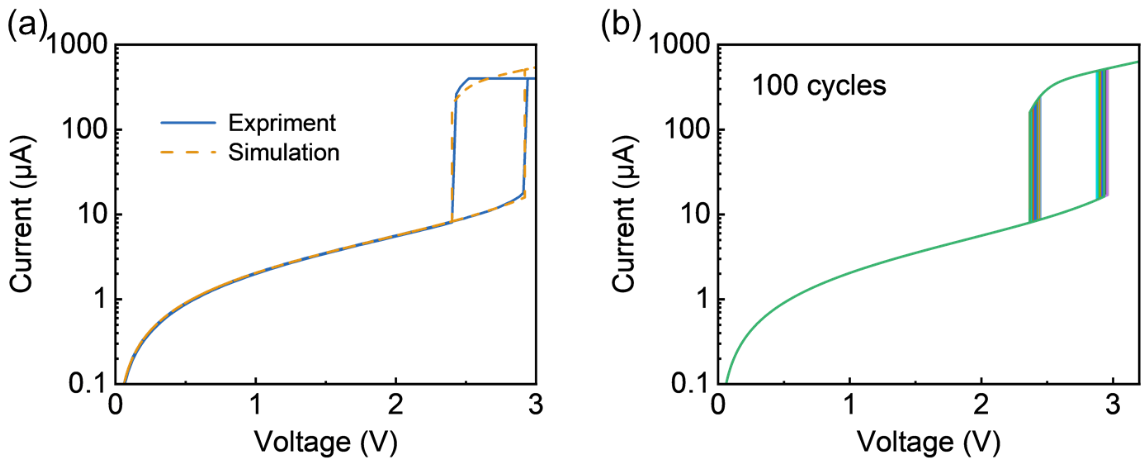

Supplementary Figure 16. The device model with the randomness of the V_{TH} and V_{Hold}. a) The empirical device model used in Text S1, which matches the experimental data well. b) Based on the empirical device model, we introduced the randomness into the V_{TH} and V_{Hold} of the device model. The I-V curve shows random fluctuations in the threshold voltages of the model at 100 DC scan cycles.

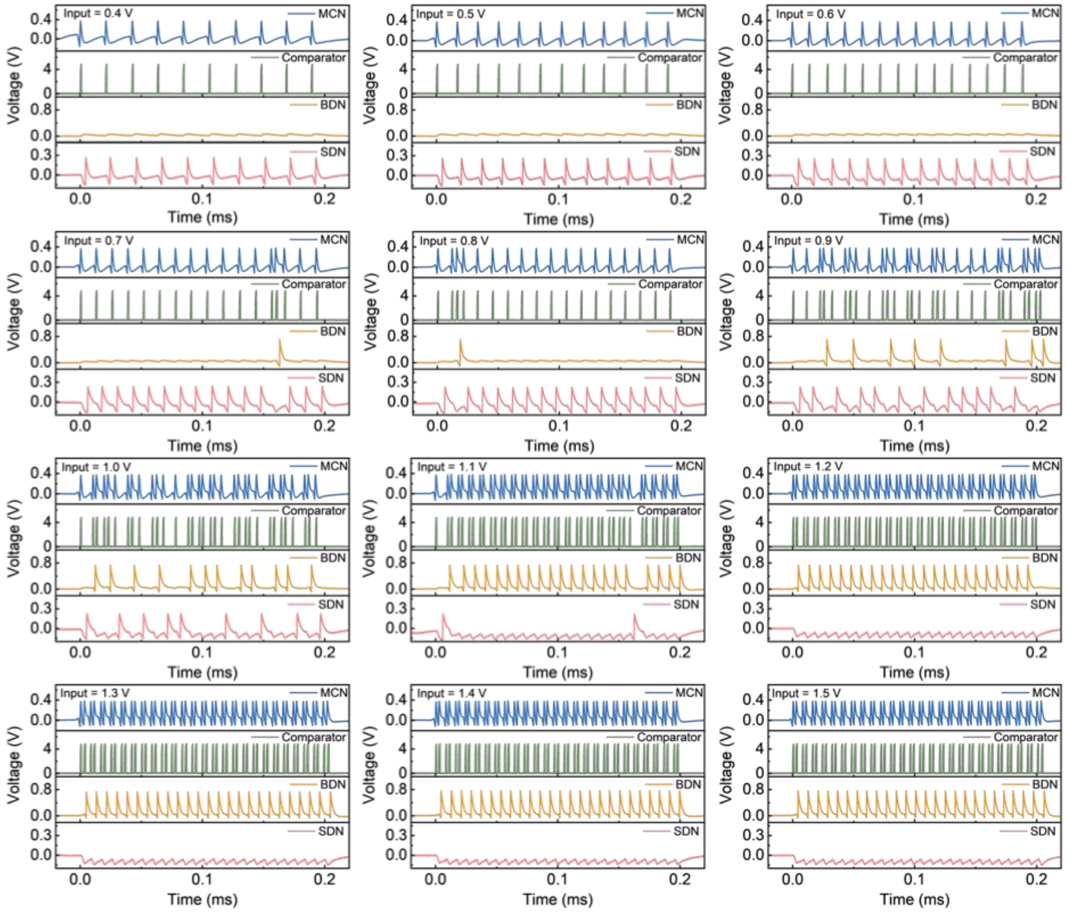

Supplementary Figure 17. The selective communication output of the neural circuits under different input voltage.

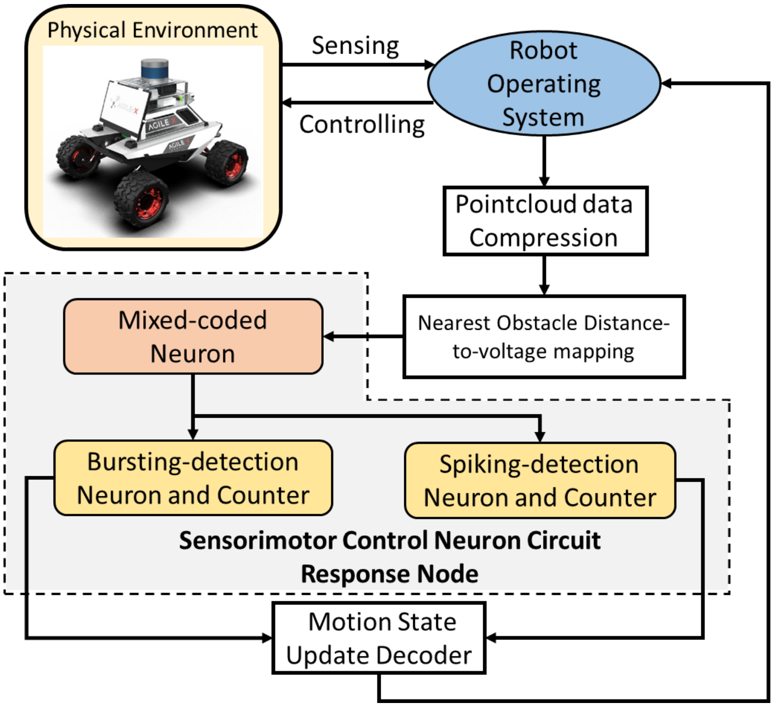

Supplementary Figure 18. Flow chart of robot obstacle avoidance behavior control. The whole experiment is conducted in the physical environment. Robot Operating System is the intermediate of Sensing and Controlling. After receiving and compressing the Point Cloud Data from lidar, the distance of the nearest obstacle is interpreted as the voltage input to the sensorimotor control neural circuit response node, which involves a mixed-coded neuron encoder, bursting-detection neuron and spiking-detection neuron decoder. The output firing frequencies of the sensorimotor control neural circuit response node are then calculated and converted to a motion direction set including angular and linear velocity, which is then delivered to the Motion Controller.

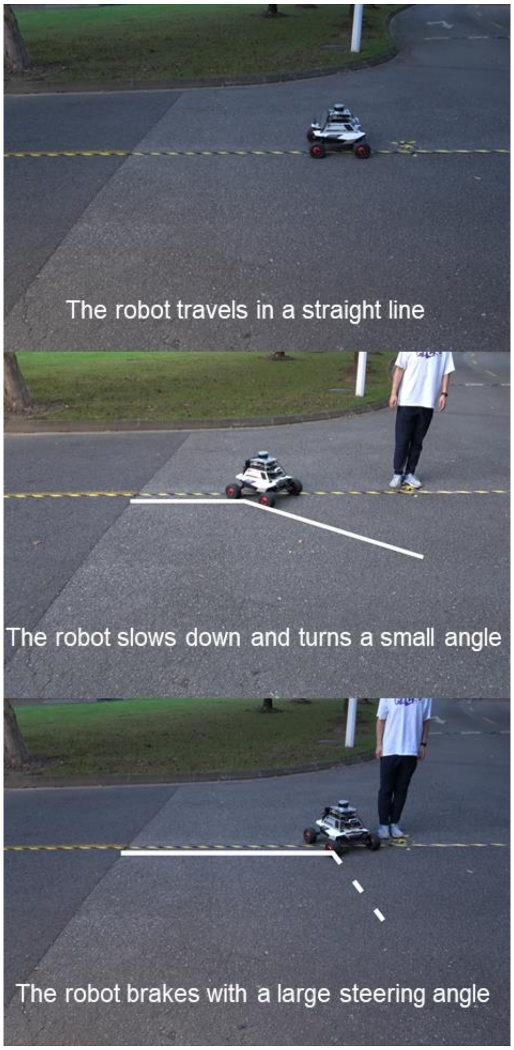

Supplementary Figure 19. A rendering of the robot's obstacle avoidance. When pedestrians (obstacles) suddenly appear in different positions of the robot's visual field, the robot produces different obstacle avoidance behaviors. If a pedestrian comes very close to the car, the robot stops and turns at a large angle. When a pedestrian comes close (far) away from the car, the car slows down and turns at a small (no) angle.

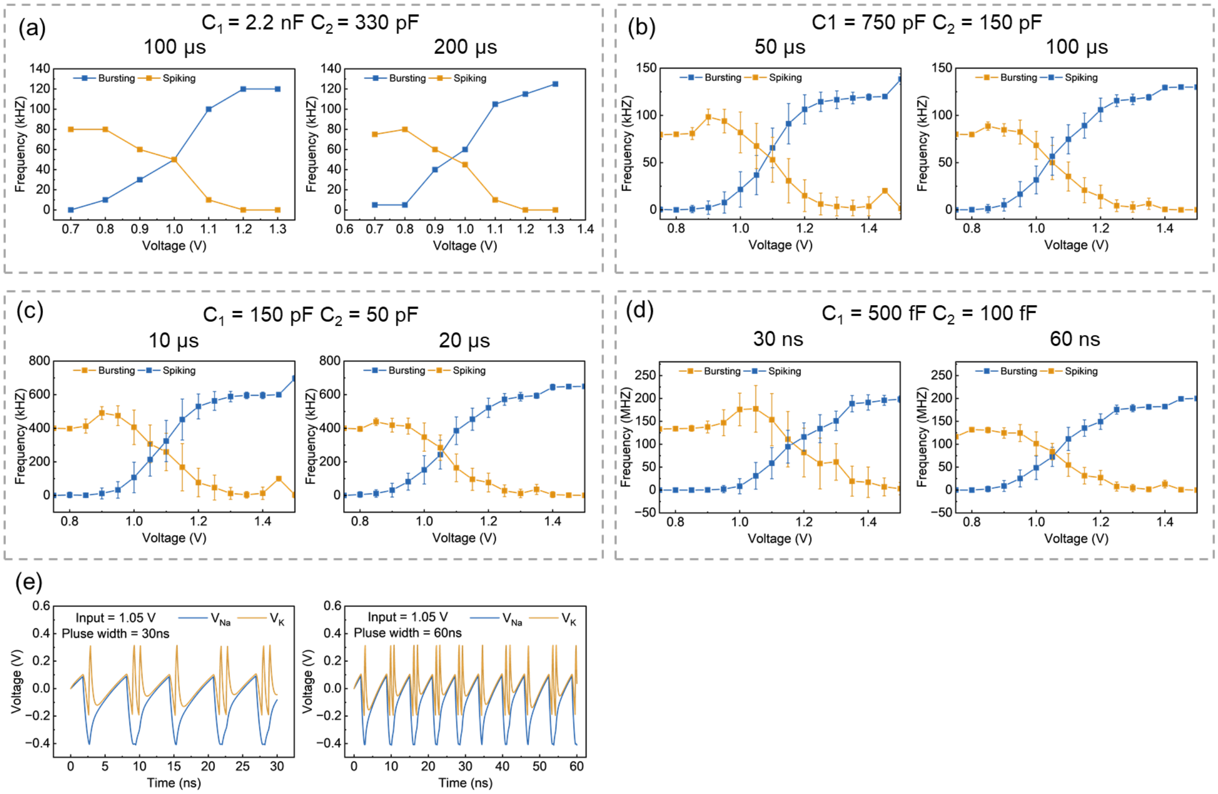

Supplementary Figure 20. Simulated latency scaling of the memristive H-H neurons. The minimum latency of the circuit is determined by the capacitances in the circuit. We define the pulse width when the input and output curves of the circuit begin to have partial distortion as the minimum latency of the circuit. a) According to the statistics of the data in Figure S15, the curve has several abnormal points and large errors under the input pulse width of 100 μs (only the number of spikes or bursts under the first 100μs of pulse application was counted) compared to 200 μs but still satisfies the rule on the whole. In this case we can consider the minimum delay of the neuron circuit to be 100μs. Similarly, we can reduce the minimum latency of the circuit to 50μs and 10μs by reducing C_1/C_2 to (b) 750 pF/150 pF and(c) 150 pF/50 pF in the simulation. d) When the capacitance is reduced to C_1 = 500 fF and C_2 = 100 fF, the minimum delay of the circuit can be reduced to ~30 ns, indicating a fast reflex-like response. e) The output of the neuron circuit under the circuit parameters in D. It is worth noting that in the simulation process, we use an empirical model to simulate the electrical characteristics of the device, so the influence of the device switching speed on the circuit output is not considered. In fact, the device switching speed can be as low as 1 ns[2], and under

ideal conditions, the output of the neuron circuit at an input of 30 ns pulse width can still observe the probabilistic transition behavior. The error bars represent the standard deviation (σ) of each group.

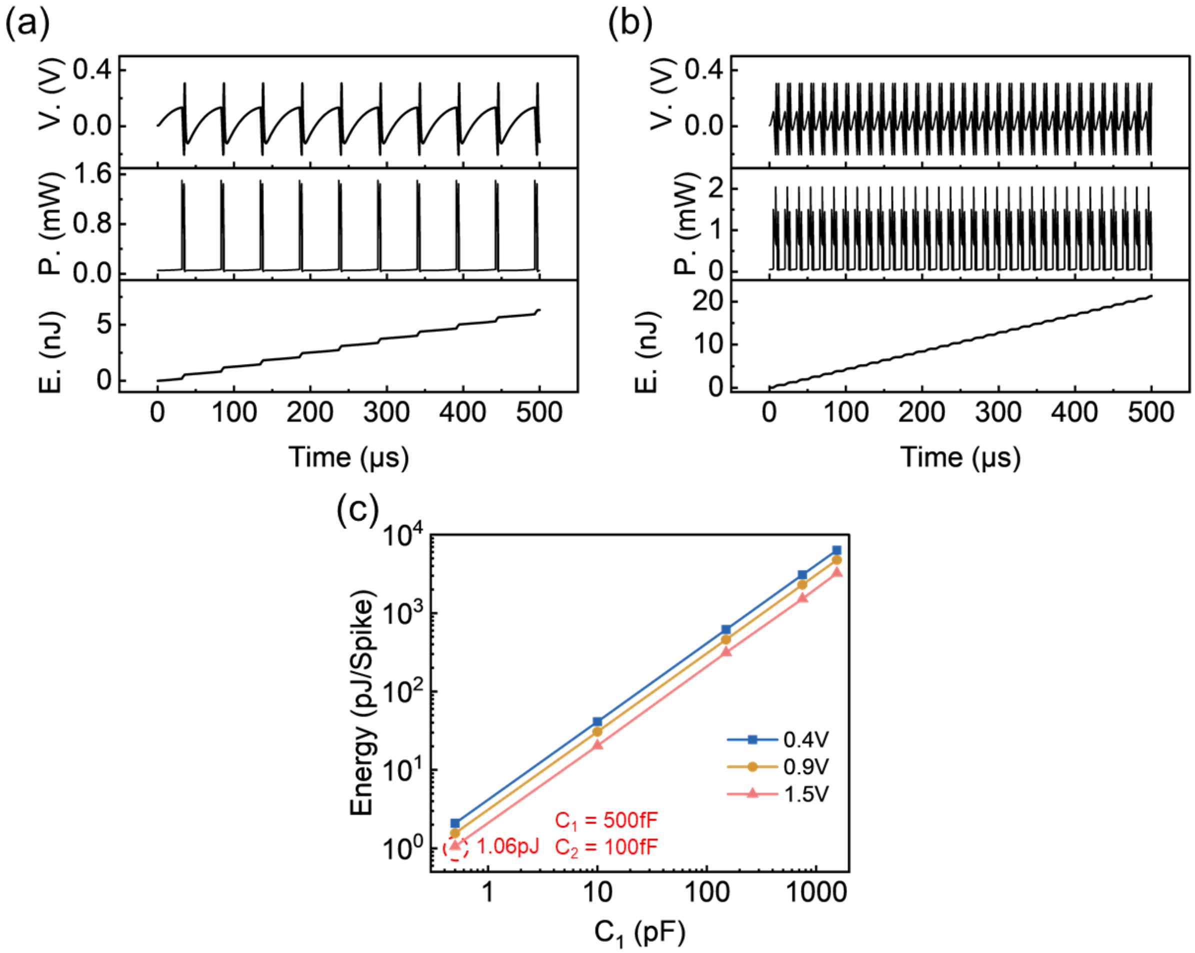

Supplementary Figure 21. Simulated energy scaling of the memristive H-H neurons. a) Output, power and energy consumption of the circuit when C_1 = 1.55 nF, C_2 = 330 pF and input = 0.4 V. The energy consumption is obtained by integrating the power provided by the two voltage sources E_1 and E_2. The energy consumption required to generate each spike is obtained by dividing the total energy consumption during the pulse time by the number of pulses. b) Output, power and energy consumption of the circuit when C_1 = 1.55 nF, C_2 = 330 pF and input = 1.5 V. c) Simulated energy of each spike vs. capacitances, showing a nearly linear scaling with the capacitor values. In the process of simulating energy scaling, the relationship C_1:C_2 = 5 is always maintained to ensure the correctness of the data. It is worth noting that the energy consumption brought by each spike is not only related to the capacitance value, but also related to the input of the circuit. When the input is 0.4 V, the circuit is just able to generate the spiking output, and the frequency is low, so the integration process before each spike is issued is long. It results in a large energy consumption, which is the upper limit of the output spike energy consumption of the whole circuit (blue line). When the output is 0.9 V, it is the critical value for the bursting output of the circuit. At this

time, the output of the circuit is all spiking mode with high frequency, and the energy consumption of a single spike is reduced compared with that of 0.4 V input, which is the lower limit of energy consumption of spiking output (yellow line). When the input is 1.5 V, the neuron circuit has all been bursting output, and the energy consumption of each spike is further reduced in this case. The energy consumption of each spike at this time is the lower limit of energy consumption when the circuit is bursting output (pink line). When the circuit is fired in mixed mode, the energy consumption of a single spike is in the range between the yellow and pink lines. As the capacitance decreases, the spike energy consumption also decreases. When the capacitance is reduced to a ~fF level (C_1 = 500 fF, C_2 = 100 fF), the energy consumption of a single pulse can be reduced to 1.06 pJ/Spike.

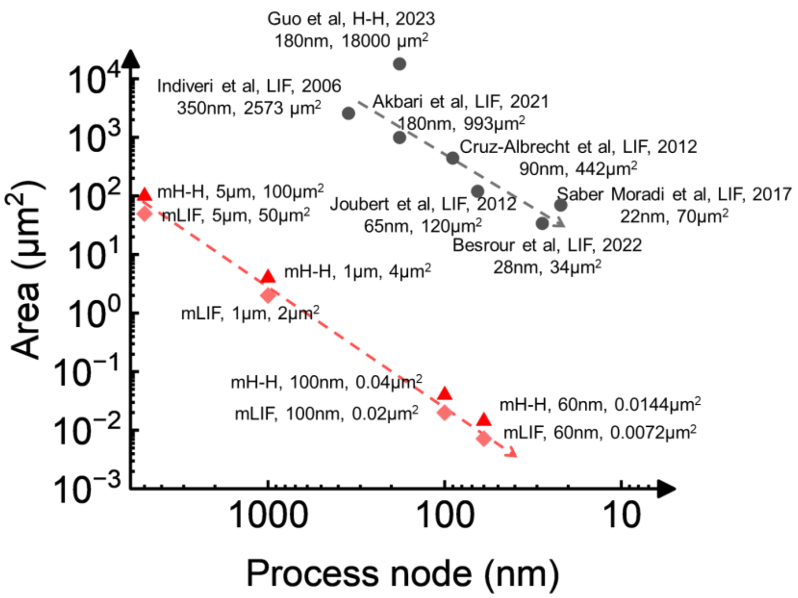

Supplementary Figure 22. Comparison of the scalability of the memristor-based on-chip LIF / H-H neuron circuits with CMOS technologies. If we use resistive variable devices (RRAM) to replace resistors, and external large capacitors can be replaced with device parasitic capacitance and construct on-chip integrated H-H neuronal circuits, the overall area of the H-H neuronal circuit can be estimated as 4A μm² and LIF as 2A μm², where A represents the effective area of RRAM and TS devices[3-9].

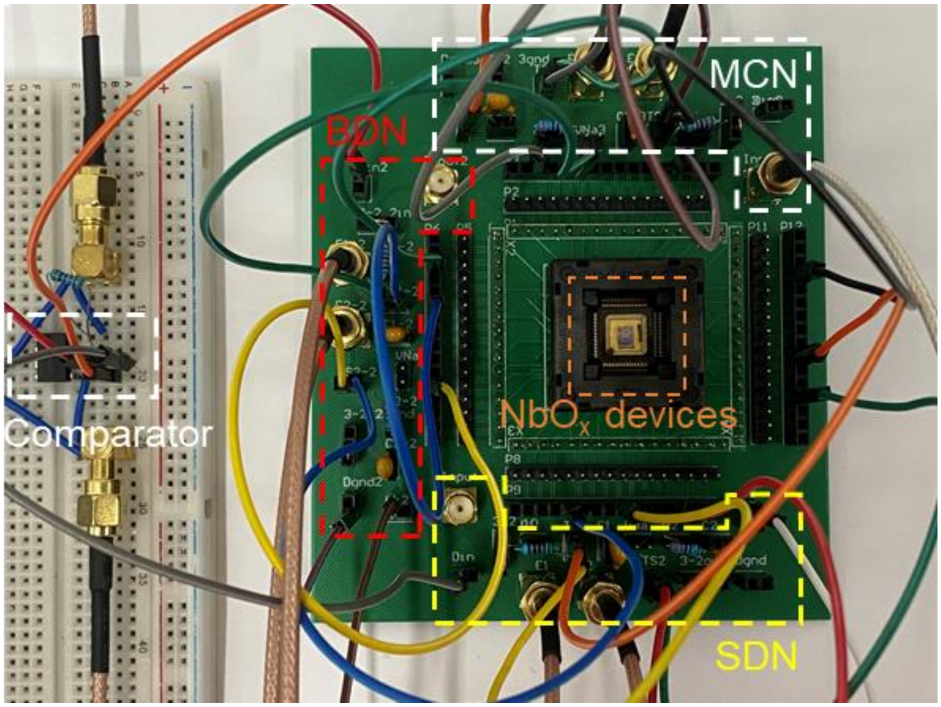

Supplementary Figure 23. The SCNC was constructed via a printed circuit board (PCB) and a breadboard. The SCNC contained MCN (white part), BDN (red part) and SDN (yellow part) and a comparator (white part in breadboard). Each neuron circuit contains two TS devices (orange part).

Supplementary Table 1. Comparison with literature reported artificial neurons based on memristors

Neuron model	LIF	H-H	H-H	H-H	H-H
Article	10-12	13	14	15	This work
Functional material	NbO_x	NbO_x	VO_2	VO_2	NbO_2
Firing behaviors achieved	4 (All-or-nothing, refractory period, class 1 excitable, tonic spiking)	4 (All-or-nothing, refractory period, tonic spiking, tonic bursting)	23	5 (All-or-nothing, refractory period, tonic spiking, tonic bursting, spike frequency adaptation)	24 (23 kinds of firing behaviors in [5] and probability switch)
Application	Yes	No	No	Yes	Yes
Firing behaviors utilized in the application	1 (class 1 excitable)	-	-	1 (spike frequency adaptation)	5 (tonic spiking, tonic bursting, probability switch, resonator, integrator)

Supplementary Table 2. Circuit parameters used for achieving 23 firing behaviors of the H-H neuron

Firing mode	$R_1(\Omega)$	$C_0(F)$	$C_{in}(F)$	$R_2(\Omega)$	$C_1(F)$	$C_2(F)$	$E_1(V)$	$E_2(V)$
All or nothing	20k	-	-	3.3k	1n	330p	-1.6	1.6
Refractory periods	20k	-	-	3.3k	1.33n	330p	-1.6	1.6
Class 1 excitable	20k	-	-	3.3k	1n	330p	-1.59	1.59
Class 2 excitable	20k	-	-	3.3k	1n	1n	-1.61	1.61
Tonic spiking	20k	-	-	3.3k	1n	330p	-1.59	1.59
Tonic bursting	20k	-	-	3.3k	1n	330p	-1.59	1.59
Spike frequency adaptation (tonic)	20k	-	-	3.3k	1u	2.2n	-1.9	2.3
Spike frequency adaptation (phasic)	30n	-	-	3.3k	1n	330p	-1.9	2.5
Subthreshold oscillation	20k	-	-	3.3k	1n	330p	-1.58	1.58
Spike latency	20k	-	-	3.3k	2.2n	1n	-1.9	1.9
Integrator	20k	-	-	3.3k	2.2n	330p	-1.9	2.01
Bistability	5.1k	-	-	3.3K	660p	660p	-1.85	1.8
Inhibition-induced spiking	20k	-	-	3.3k	2.2n	1n	-1.55	1.57
Inhibition-induced bursting	20k	-	-	3.3k	2.2n	20p	-1.55	1.57
Excitation block	20k	-	-	1k	1.33n	330p	-1.52	1.53
Resonator	9.4k	-	-	3.3k	2n	1n	-1.59	1.59
Phasic spiking	-	-	660p	3.3k	1n	2.2n	-1.9	2.8
Phasic bursting	-	-	2n	3.3k	1n	2.2n	-1.9	2.5
Rebound spiking	-	-	330p	3.3k	0	2n	-3.5	3.61
Rebound bursting	-	-	1n	3.3k	0	2n	-3.5	3.61
Threshold variability	-	-	1n	3.3k	0	2n	-3.5	3.61
Accommodation	-	-	330p	3.3k	2n	3n	-3	2.7
Mixed mode	50k	10n	-	3.3k	1n	1n	-2.99	3
Depolarizing after potential	10k	-	330p	3.3k	2n	330p	-1.8	1.8

Supplementary Table 3. Circuit parameters used for experiment or simulation in the neural circuits

Neuron	Firing feature	Figure No.	R_1 (kΩ)	R_2 (kΩ)	C_1 (nF)	C_2 (nF)	E_1 (V)	E_2 (V)	TS_1 (ID)	TS_2 (ID)
H-H neuron	Tonic spiking (bursting)	Fig. 2d/e	20	4	2.2	0.47	-2.72	2.51	A7 1-5-12	A7 1-3-15
MCN	Tonic spiking (bursting)	Fig. 3d / Fig. S9-10	20	4	2.2	0.47	-2.7	2.55	A7 1-5-12	A7 1-3-15
H-H neuron (Simulation)	Tonic spiking (bursting)	Fig. 4e/Fig. S18	29	4	1.55	0.33	-2.82	2.72	-	-
H-H neuron (Simulation)	Tonic spiking (bursting)	Fig. 2c / Fig. 3c/Fig. S5	24.5	4	1.55	0.33	-2.82	2.72	-	-
SDN	Resonator	Fig. 4c/d /Fig. S13	9.4	3.3	2.2	1	-1.53	1.53	A10 1-1-5	A10 1-2-9
BDN	Integrator	Fig. 4c/d /Fig. S13	25	3.3	2.2	1	-1.53	1.53	A10 1-1-5	A10 1-2-9
MCN	Tonic spiking (bursting)	Fig. 5d/Fig. S15	20	4	2.2	0.47	-1.62	1.59	B2 2-5-8	B2 2-6-11
SDN	Resonator	Fig. 5d/Fig. S15	5	6.8	2	1.47	-1.66	1.38	B2 4-4-5	B2 4-7-3
BDN	Integrator	Fig. 5d/Fig. S15	20	3.3	1.47	0.33	-1.43	1.43	B2 4-2-2	B2 4-6-2
MCN after scaling (Simulation)	Tonic spiking (bursting)	Fig.5f	24.5	4	5e-4	1e-4	-2.82	2.72	-	-
SDN after scaling (Simulation)	Resonator	Fig.5f	17	4	6e-4	2.66e-4	-1.54	1.55	-	-
BDN after scaling (Simulation)	Integrator	Fig.5f	42	2	4.3e-4	2.33e-4	-1.52	1.52	-	-

Supplementary Table 4. Comparison with literature reported artificial neurons based on CMOS

Reference	Neuron model	Technology	Energy per spike	Complexity
Besrour et al[9]	LIF	CMOS 28 nm	1.2 fJ	8T+2C
Chen et al[16]	LIF	CMOS 65 nm	4 pJ	13T+1C
Rubino et al[17]	AdExp IF	CMOS 22 nm	0.99 pJ	56T+4C
Nair et al[18]	AdExp IF	CMOS 180 nm	10 pJ	34T+3C
Guo et al[3]	H-H	CMOS 180 nm	20 pJ	4T+6R+2C
Ma et al[19]	H-H	CMOS 130 nm	170 pJ	43T
Hu et al[20]	H-H	Discrete components	-	12Ops+ 4NPNs+31R+4 C
Rutherford et al[21]	H-H	Discrete components	-	9OPs+21R+9C
This work	H-H	Discrete components	~3 nJ/1.06 pJ*	2TS+2R+2C

*: The energy of our H-H neuron circuit is about 3 nJ (@C_1 = 1.55 nF, C_2 = 330 pF) and 1.06 pJ (@C_1 = 500 fF, C_2 = 100 fF).

Supplementary Table 5. Parameters used for the SPICE model

Physical quantity	Symbols	Values	Units
Thermal capacitance	C_{th}	8.00E-17	J/K
Thermal conductance	Γ_{th}	4.00E-07	W/K
Bolzmann's constant	k	1.38E-23	J/K
Activation energy	Ea	0.26	eV
Electron charge	q	1.60E-09	C
Fitting constant	R_0	4200	Ω
Vacuum permittivity	ε_0	8.85E-12	F/m
Relative permittivity	ε_r	45	
Oxide thickness	t_{ox}	22	nm
Ambient temperature	T_{amb}	298	K

Supplementary References

1 Izhikevich, E. M., Desai, N. S., Walcott, E. C. & Hoppensteadt, F. C. Bursts as a unit of neural information: selective communication via resonance. *Trends. Neurosci.* **26**, 161-167 (2003).

2 Pickett, M. D. & Williams, R. S. Sub-100 fJ and sub-nanosecond thermally driven threshold switching in niobium oxide crosspoint nanodevices. *Nanotechnology* **23**, 215202 (2012).

3 Guo, C., Xiao, Y., Jian, M., Zhao, J. & Sun, B. Design and optimization of a new CMOS high-speed H–H neuron. *Microelectronics J.* **136**, 105774 (2023).

4 Indiveri, G., Chicca, E. & Douglas, R. A VLSI array of low-power spiking neurons and bistable synapses with spike-timing dependent plasticity. *IEEE T. Neural Networ.* **17**, 211-221 (2006).

5 Akbari, M., Hussein, S. M., Chou, T.-I. & Tang, K.-T. A 0.3-V conductance-based silicon neuron in 0.18 μm CMOS process. *IEEE T. Circuits-II: Express Briefs* **68**, 3209-3213 (2021).

6 Cruz-Albrecht, J. M., Yung, M. W. & Srinivasa, N. Energy-efficient neuron, synapse and STDP integrated circuits. *IEEE T. Biomed. Circ. S.* **6**, 246-256 (2012).

7 Joubert, A., Belhadj, B., Temam, O. & H ́eliot, R. Hardware spiking neurons design: analog or digital?, in *the 2012 International Joint Conference on Neural Networks (IJCNN)*, 1-5 (IEEE, 2012).

8 Moradi, S., Bhave, S. A. & Manohar, R. Energy-efficient hybrid CMOS-NEMS LIF neuron circuit in 28nm CMOS process, in *the 2017 IEEE Symposium Series on Computational Intelligence (SSCI)*, 1-5 (IEEE, 2017).

9 Besrour, M. *et al.* Analog spiking neuron in 28 nm CMOS, in *the 2022 20th IEEE Interregional NEWCAS Conference (NEWCAS)*, 148-152 (IEEE, 2022).

10 Zhang, X. *et al.* An artificial spiking afferent nerve based on Mott memristors for neurorobotics. *Nat. Commun.* **11**, 51 (2020).

11 Chen, P. *et al.* High-yield and uniform NbOx-based threshold switching devices for neuron applications. *IEEE T. Electron. Dev* **69**, 2391-2397 (2022).

12 Zhang, X. *et al.* An Artificial Neuron Based on a Threshold Switching Memristor. *IEEE Electron Device Letters* **39**, 308-311 (2018).

13 Pickett, M. D., Medeiros-Ribeiro, G. & Williams, R. S. A scalable neuristor built with Mott memristors. *Nat. Mater.* **12**, 114-117 (2013).

14 Yi, W. *et al.* Biological plausibility and stochasticity in scalable VO_2 active memristor neurons. *Nat. Commun.* **9**, 4661 (2018).

15 Xu, Y., Gao, S., Li, Z., Yang, R. & Miao, X. Adaptive Hodgkin–Huxley neuron for retina‐inspired perception. *Adv. Intell. Syst.* **4**, 2200210 (2022).

16 Chen, X. *et al.* CMOS-based area-and-power-efficient neuron and synapse circuits for time-domain analog spiking neural networks. *Applied Physics Letters* **122**, 4102 (2023).

17 Rubino, A., Payvand, M. & Indiveri, G. Ultra-low power silicon neuron circuit for extreme-edge neuromorphic intelligence, in *the 2019 26th IEEE International Conference on Electronics Circuits and Systems (ICECS)*, 19296947, 458-461 (IEEE, 2019).

18 Nair, M. V. & Indiveri, G. An ultra-low power sigma-delta neuron circuit, in *the 2019 IEEE International Symposium on Circuits and Systems (ISCAS)*, 18815532, 1-5 (IEEE, 2019).

19 Ma, Q., Haider, M. R., Shrestha, V. L. & Massoud, Y. Bursting Hodgkin–Huxley model-based ultra-low-power neuromimetic silicon neuron. *Analog Integrated Circuits and Signal Processing* **73**, 329-337 (2012).

20 Hu, X. & Liu, C. Dynamic property analysis and circuit implementation of simplified memristive Hodgkin–Huxley neuron model. *Nonlinear Dynamics* **97**, 1721-1733 (2019).
21 Rutherford, G. H., Mobille, Z. D., Brandt-Trainer, J., Follmann, R. & Rosa, E. Analog implementation of a Hodgkin–Huxley model neuron. *American Journal of Physics* **88**, 918-923 (2020).
