# Supplementary material for: Firing feature-driven neural circuits with scalable memristive neurons for robotic obstacle avoidance
Source: Nat Commun. 2024 May 21;15:4318. doi: 10.1038/s41467-024-48399-7 (PMC11109161; doi:10.1038/s41467-024-48399-7)
Supplement: Supplementary file 3 — Description of Additional Supplementary Files [file 41467_2024_48399_MOESM3_ESM.pdf]

Description of Additional Supplementary Files

Supplementary Movie 1. Response of the robot when there is no obstacle on the current path.
Supplementary Movie 2. Response of the robot when the obstacle is at a moderate distance.
Supplementary Movie 3. Response of the robot when the obstacle is in close proximity.
Supplementary Movie 4. The continuous obstacle avoidance behavior of the robot

Supplementary Code 1. Python scripts implementing SCNC model for robot obstacle avoidance control
